# Regulation of the host immune system by helminth parasites

**DOI:** 10.1016/j.jaci.2016.07.007

**Published:** 2016-09

**Authors:** Rick M. Maizels, Henry J. McSorley

**Affiliations:** a Wellcome Trust Centre for Molecular Parasitology, Institute of Infection, Immunity and Inflammation, University of Glasgow, Glasgow, United Kingdom; b Centre for Inflammation Research, University of Edinburgh, Queen's Medical Research Institute, Edinburgh, United Kingdom

**Keywords:** Allergy, infection, pathology, therapy, tolerance, Breg, Regulatory B, DC, Dendritic cell, Foxp3, Forkhead box protein 3, Treg, Regulatory T

## Abstract

Helminth parasite infections are associated with a battery of immunomodulatory mechanisms that affect all facets of the host immune response to ensure their persistence within the host. This broad-spectrum modulation of host immunity has intended and unintended consequences, both advantageous and disadvantageous. Thus the host can benefit from suppression of collateral damage during parasite infection and from reduced allergic, autoimmune, and inflammatory reactions. However, helminth infection can also be detrimental in reducing vaccine responses, increasing susceptibility to coinfection and potentially reducing tumor immunosurveillance. In this review we will summarize the panoply of immunomodulatory mechanisms used by helminths, their potential utility in human disease, and prospective areas of future research.

*Discuss this article on the JACI Journal Club blog:* *www.jaci-online.blogspot.com*.

Helminths are highly prevalent metazoan worm parasites, which have evolved a spectrum of sophisticated means to regulate and evade the host immune system.^1^ Helminths appear to act as successful xenotransplants into the mammalian body, neutralizing immune pathways that would otherwise expel them and resetting the thresholds of immune reactivity.^2^ In so doing, they also dampen responses to unrelated bystander specificities, such as allergens and autoantigens, in a manner that might in fact benefit the host.^1^, ^3^

Only a dozen or so species of helminths are widespread in human subjects, but together, they infect some 2 billion persons, nearly one third of the human population.^4^ Their extraordinary prevalence bears witness to their success at defeating host defenses and suggests we have much to learn from how these parasites modulate our own immune system.

Although helminths establish in a range of tissue and intestinal niches, in nearly all cases they do not multiply within the host but produce eggs or larvae to infect new hosts; hence they tend to establish stable chronic infections that can endure for surprisingly long (up to 20 years) in an individual host. In this setting almost every facet of the immune system is modified or even recalibrated, with infected subjects displaying a state of immune hyporesponsiveness that can be considered a form of immunologic tolerance.^5^, ^6^, ^7^

## Immunologic tolerance in human helminth infections

Immunologic hyporesponsiveness in helminth infections was first seen through muted parasite antigen-specific T-cell responses, from patients' PBMC cultures.^7^, ^8^, ^9^, ^10^ In particular, specific unresponsiveness was seen in asymptomatic carriers rather than those with progressive pathologic manifestations, such as elephantiasis. Furthermore, anthelminthic drug clearance of parasites from hyporesponsive carriers resulted in a recovery of antigen-specific responses, suggesting that they were actively inhibited by the presence of helminths.^10^, ^11^ In addition, T cells from helminth-infected asymptomatic human subjects show skewed cytokine profiles, favoring IL-4 over IL-17 and IFN-γ^10^ and with more conspicuous IL-10 and TGF-β components.^12^, ^13^ In contrast, in patients in whom symptomatic disease develops, there is a failure of tolerance, allowing T_H_1 and T_H_17 responses to surface and mediate significant pathology in infected tissues.^14^, ^15^

In the immune system homeostatic tolerance to self-antigens and harmless environmental antigens (including commensal bacteria and food components) is primarily maintained by an immunosuppressive T-cell subset, the regulatory T (Treg) cell.^16^ As discussed below, a strong link has emerged between long-term helminth infection and Treg cell activity, particularly in the asymptomatic or hyporesponsive state.^17^, ^18^, ^19^

Immune downregulation by helminths further extends into many local and systemic settings, with modulation of responses to a variety of unrelated bystander specificities.^3^ One example is that polyclonal immune responses to childhood vaccines can be compromised in heavily infected subjects.^20^ In addition, helminths can undermine host defenses against other major pathogens, such as *Mycobacterium tuberculosis*.^3^, ^21^ In the case of malaria, however, the consequences of helminth infection are more nuanced, with evidence of increased susceptibility combined with moderated inflammatory responses and hence attenuated disease severity.^22^, ^23^

The delicate balance between inflammation and immune regulation is exemplified in cysticercosis, a neurological pathology caused by inflammatory responses to *Taenia solium* cysts.^24^ It is well recognized that pathologic inflammation can be dampened by immunoregulatory mechanisms that might underpin the asymptomatic phase of disease.^25^ However, patients with the most highly disseminated infections actually show the greatest degree of immunoregulation, with increased IL-10 levels, decreased T_H_1 and T_H_2 cytokine levels, and a trend for increased Treg cell numbers.^26^ Thus the immune response to *T solium* infection is finely poised: strong immunomodulation leads to dissemination of the parasite, whereas failure to regulate inflammation causes seizures and death.

Helminth parasites clearly establish hyporesponsiveness in the naive adult in model systems, but in endemic settings it is common for offspring to be born to infected mothers, become infected at a very early age, or both. Maternal infection boosts tolerance in the newborn, so that offspring of Haitian mothers with the filarial infection *Wuchereria bancrofti* were 2- to 3-fold more likely to become infected themselves while showing a lower level of T-cell reactivity to parasite antigens than children of uninfected mothers.^27^ In a remarkable study in the Cook Islands, it was confirmed that even at 17 years of age, subjects born to infected mothers mounted substantially weaker T-cell responses to parasite antigens.^28^ Hence in the endemic setting it seems likely that many subjects experience *in utero* tolerization to parasite antigens. Furthermore, prenatal exposure also affects bystander reactivities both in human subjects^29^ and in experimental models, such as airway allergy.^30^ As will be discussed further, anti-inflammatory effects of helminth infection are observed not only in the setting of allergy but also in the context of autoimmunity and transplantation reactions.^2^, ^31^, ^32^

## Treg cells in helminth infection: from the field to the laboratory

A key association has emerged between helminth infection and expansion of regulatory cell populations, most importantly the Treg cell subset.^17^, ^18^, ^33^ In human subjects Treg cells expressing the transcription factor forkhead box protein 3 (Foxp3) are more numerous and more active in helminth-infected subjects but decrease after anthelmintic chemotherapy.^19^, ^34^, ^35^ In filarial infections patients with pathologies, such as elephantiasis and hyperreactive onchocerciasis, show diminished Treg cell levels compared with those in unresponsive asymptomatic carriers, supporting the argument that the Treg cell compartment both maintains tolerance and prevents pathology in these infections.^15^, ^36^, ^37^ Likewise, in highly prevalent soil-transmitted intestinal nematode infections, a similar profile of increased Treg cell activity, immunosuppressive cytokine production, and antigen hyporesponsiveness is evident.^38^, ^39^ Mechanistically, multiple pathways are implicated in the downregulation of human responses to helminths, involving the cytokines IL-10 and TGF-β, and cell-surface interactions through cytotoxic T lymphocyte–associated antigen 4 and programmed death-1.^19^, ^38^, ^39^, ^40^

In human subjects the activity of Treg cells and production of IL-10 correlate closely with an isotype switch from the proallergic/inflammatory IgE to the noninflammatory IgG_4_^41^; Foxp3^−^ T_R_1 cell are the predominant source of IL-10,^36^ although Foxp3^+^ Treg cells are also present, and both contribute to driving IgG_4_ in human subjects.^42^ Serum IgG_4_ is largely composed of mixed dimers. Because the heavy chains lack linking disulfide bonds, they exchange with other IgG_4_ molecules; such mixed molecules are functionally monovalent and noninflammatory.^43^ Drug treatment of patients resulted in sharp decreases in circulating IgG_4_ levels, again arguing that parasites press the host immune system to favor this isotype.^44^

The causal links between helminth infections and Treg cells have now been established in both directions. First, certain helminths directly drive Treg cell responses from the host^45^ or do so indirectly through inducing host cells to produce TGF-β, a key cytokine that promotes regulatory cell function.^46^ Hence the expansion of Treg cells is not simply a corollary of the host inflammatory response that must accompany it to prevent overreaction.

Second, Treg cells are essential for parasites to survive in the immunocompetent host because their depletion in mouse model systems results in clearance of the infection,^47^, ^48^, ^49^ whereas expansion of Treg cells through IL-2 administration renders mice more susceptible.^49^ Interestingly, in the mouse model of filariasis, Treg cells establish hyporesponsiveness in the effector population, so that clearance of tissue-dwelling parasites requires not only ablation of Treg cells but also restimulation of the effector population.^50^, ^51^

The effects of Treg cells in murine helminth infections also mirror those in human subjects in other ways. For example, Treg cells are instrumental in attenuation of allergy in mice infected with gastrointestinal nematodes^52^ or schistosomes.^53^ They also play a vital role protecting the host from pathology because Treg cell depletion can exacerbate inflammatory responses with lethal results.^48^, ^49^, ^54^ Thus although partial Treg cell depletion can strengthen the T_H_2 response required for parasite expulsion, in their total absence an inflammatory storm prevails, preventing a coherent protective immune response.^49^

Treg cells are also implicated in the weakened defenses against other parasites, and in human subjects *in vitro* T-cell proliferative responses to BCG and malaria are attenuated in helminth-infected patients but recover if Treg cells are removed from the test cultures.^55^ Similarly, BCG vaccination of helminth-infected subjects elicits poor inflammatory cytokine responses to purified protein derivative antigen in contrast to significant TGF-β production; anthelmintic treatment reverses this scenario, suggesting that interference with vaccine responses might be due to the presence of immunosuppressive cytokines.^56^ Supporting this, a recent study reported that tuberculosis-infected migrants in the United Kingdom who were coinfected with helminths had higher Treg cell frequencies than those with tuberculosis alone but that anthelmintic treatment decreased Treg cell numbers while increasing T_H_1 effector populations.^57^

## Regulatory B cells, dendritic cells, and macrophages in helminth infection

Often overshadowed by their T-cell counterparts, regulatory B (Breg) cells are also crucially important in control of the immune response during helminth infection.^58^ B cells from *Heligmosomoides polygyrus*–infected mice can suppress experimental autoimmune encephalomyelitis and airway allergy when transferred to recipient mice.^59^ Similarly, airway allergy can be suppressed by B cells from *Schistosoma mansoni*–infected mice, directly through their production of IL-10 and indirectly by enhancing Treg cell activity.^60^ Importantly, the latter study found similar Breg phenotype cells in schistosome-infected human subjects. High numbers of functional IL-10 producing Breg cells were also found in patients with multiple sclerosis protected from relapse after acquiring intestinal helminth infection compared with otherwise comparable uninfected patients.^61^ Along with Treg cell, Breg cells are strongly implicated in the development of tolerance to allergens,^62^ and strategies to encourage their expansion could increase the efficacy of allergen-specific immunotherapy.

Dendritic cells (DCs) in patients with helminth infections have been widely investigated for their propensity to induce T_H_2 responses in distinction to microbially stimulated DCs, which effectively drive T_H_1 and T_H_17 outcomes.^63^, ^64^ As yet, how DCs recognize the presence of helminths is unresolved, although certain key intracellular signals, such as the Kruppel-like factor 4 (KLF4), are now known to be essential for DCs to adopt the pro-T_H_2 phenotype^65^; beyond this stage, the mechanisms through which DCs instruct T_H_2 development are similarly opaque but are likely to include surface interactions, such as OX40/OX40 ligand costimulation.^66^

The question of whether DCs in helminth-infected mice are more tolerogenic and contribute to the expansion of Treg cells *in vivo* is also of great interest. DCs recovered from helminth-infected mice show altered phenotypes with, in the case of *H polygyrus* infection, expansion of CDllc^lo^CD103^−^ DCs, which are preferential inducers of Foxp3^+^ Treg cells *in vitro*. In contrast, CD11c^hi^ DCs induced stronger effector responses. In CD11c^DTR^ mice diphtheria toxin administration depleted only the CD11c^hi^ subset, greatly diminishing the T_H_2 response, but spared the CD11c^lo^ population and the Treg cell response they induced.^67^ In other studies intestinal DCs from mice infected with the same parasite were able, when transferred into recombination-activating gene–deficient mice, to protect recipients from T cell–mediated colitis.^68^

A more reductionist approach has tested DCs differentiated *in vitro* from bone marrow precursors with various helminth products before appraising their ability to induce regulatory cytokines or cells from T cells or from mice receiving a bolus of pulsed DCs. A recurrent finding in these studies has been that helminth antigens (eg, secreted products or egg extracts) block the Toll-like receptor–stimulated pathway that leads to IL-12 production and T_H_1 induction.^69^, ^70^, ^71^ In terms of *in vivo* immunoregulation, DCs pulsed with *Hymenolepis diminuta* antigens are able to downmodulate dinitrobenzene sulfonic acid colitis in recipient mice, and CD4^+^ T cells from those recipients can be further transferred to new hosts and protect against colitis, requiring IL-10 production for their effect.^72^ A substantial range of different helminth molecules have now been reported to modulate DC reactivity and function, as recently reviewed,^64^ promising a more mechanistic understanding in the near future. In particular, the *S mansoni* secreted protein ω-1, a glycosylated T2 ribonuclease, is the first helminth-derived molecule in which the mechanism of action of DCs has been characterized. This glycoprotein is taken up by mannose receptor binding to the glycan side chains, and once inside the cell, its ribonuclease activity degrades host mRNA, ablating IL-12 production and encouraging T_H_2 differentiation.^73^

Macrophages in patients with helminth infection are profoundly altered in their profile, adopting an alternatively activated phenotype (also termed M2) driven by the type 2 cytokines IL-4 and IL-13 and adopting a pattern of gene expression, metabolism, and function markedly different from that of classically activated (M1) macrophages, which respond to microbial stimulation through Toll-like receptors.^74^ Signature protein products of the M2 macrophage include arginase-1, RELM-α, and the chitinase-like molecule Ym1.^75^ M2 macrophages are required for effective immunity to some parasites (including *H polygyrus*^76^, ^77^ in an arginase-1–dependent manner) and are instrumental in repair and resolution of tissue damage caused, for example, by migratory helminths.^78^ In this context helminth-stimulated macrophages adopt an anti-inflammatory role with immunosuppressive characteristics, for example inhibiting T-cell proliferation,^79^ in part through expression of programmed death ligand 1^80^ and enhancement of Treg cell differentiation through vitamin A production by retinal dehydrogenase.^81^

## Protection from allergy, autoimmunity, and allograft rejection

To investigate the immunomodulatory pathways used by helminths, it is useful to consider the responses involved in their ejection as the most likely targets for modulation. For example, in the field of virology, the class I MHC presentation pathway is crucial to orchestration of a productive antiviral CD8 T-cell response: for almost every step in this pathway, a viral immunomodulator can be found that interferes in its normal functioning.^82^ Likewise, as the field of immunoparasitology matures, it is clear that parasites have evolved strategies to modulate, subvert, or evade each component of the immune response which might be capable of eliminating them. Because it is often difficult to assess the importance of immune pathways while under active suppression during parasitic infection, models of immunopathology have been useful tools to assess modulation mediated by parasites or their products.

It has been noted since 1968 that inflammatory disorders, such as arthritis, are much less frequent in low-income countries with high levels of parasite infection.^83^ Subsequent studies have further established a reciprocal link between endemic helminth infection and reduced prevalence of allergic reactivity and autoimmune antibodies, as well as increases in these immunologic indicators after anthelmintic treatment.^84^, ^85^, ^86^ The downmodulation of immune dysfunction in the presence of helminths depends on the exact parasite species in question, as well as the intensity of infection,^87^, ^88^ but has been reported across the spectrum of tropical environments using a range of different approaches (reviewed by McSorley and Maizels^1^). It is likely that helminths impact at 2 levels: (1) modifying the level of host reactivity during development of the infant immune system and (2) dampening immune responses in mature subjects who might be exposed to helminths for the first time in adult life.^89^ It is the latter setting that led to the proposal that helminths or their products could be used as therapies for inflammatory diseases in the parasite-free developed world.^90^

In settings of both allergy and autoimmunity, attenuation of reactivity has been linked to downmodulatory cytokine production, in particular IL-10 responses.^84^, ^86^ Among the most remarkable studies has been that of a cohort of patients with multiple sclerosis in Argentina who unintentionally acquired gastrointestinal helminth infections, with subsequent increased TGF-β and IL-10 levels and higher Treg and Breg cell activity. Strikingly, the infected patients enjoyed clinical remission from symptomatic disease,^61^, ^91^ but those subjects given anthelmintic treatment experienced loss of regulatory cytokines and relapse of disease.^92^

Experimental data echo and extend these findings in laboratory models of allergic and autoimmune pathology.^1^, ^33^, ^93^, ^94^ Helminth-infected mice are less susceptible to airway inflammation after allergen sensitization, and Treg cells from these mice can confer protection against allergy when transferred to naive animals.^52^, ^53^ Moreover, Breg cells,^59^, ^95^ helminth-stimulated DCs,^68^ and regulatory macrophages^96^ are each able, in different settings, to confer protection against pathology in recipient mice.

Reports such as these have fueled interest in the administration of live helminths as therapies for a range of inflammatory conditions from allergy, autism, autoimmunity, and colitis.^90^ After some promising early studies,^97^ more recent trials have not proved significant benefit.^98^, ^99^ A number of reasons might underpin the perceived lack of efficacy of live helminth therapy.^94^ For example, the human response to helminths is spectral, and only a subset might gain benefit from live infection; each helminth species inhabits a particular anatomic niche that might or might not affect the site of inflammatory disease; the dynamics of any protective effect in terms of parasite dose and duration of infection are unknown; and therapy of an established inflammatory disorder might require a particularly high parasite load or long-term infection. For each of these reasons, a more analytic approach of identifying immunomodulatory mechanisms and molecular mediators from helminths is advocated as the best strategy for developing new therapies inspired by the immunosuppressive capacities of parasites.^100^, ^101^, ^102^ By this means, individual molecular products can be assessed, validated, and developed as defined pharmaceuticals, which can then be delivered in a manner most consistent with the indication in question. Most significantly, this approach separates benefit from harm and removes the need to introduce a potentially pathogenic parasite in the treatment of disease.

**Fig 1:**
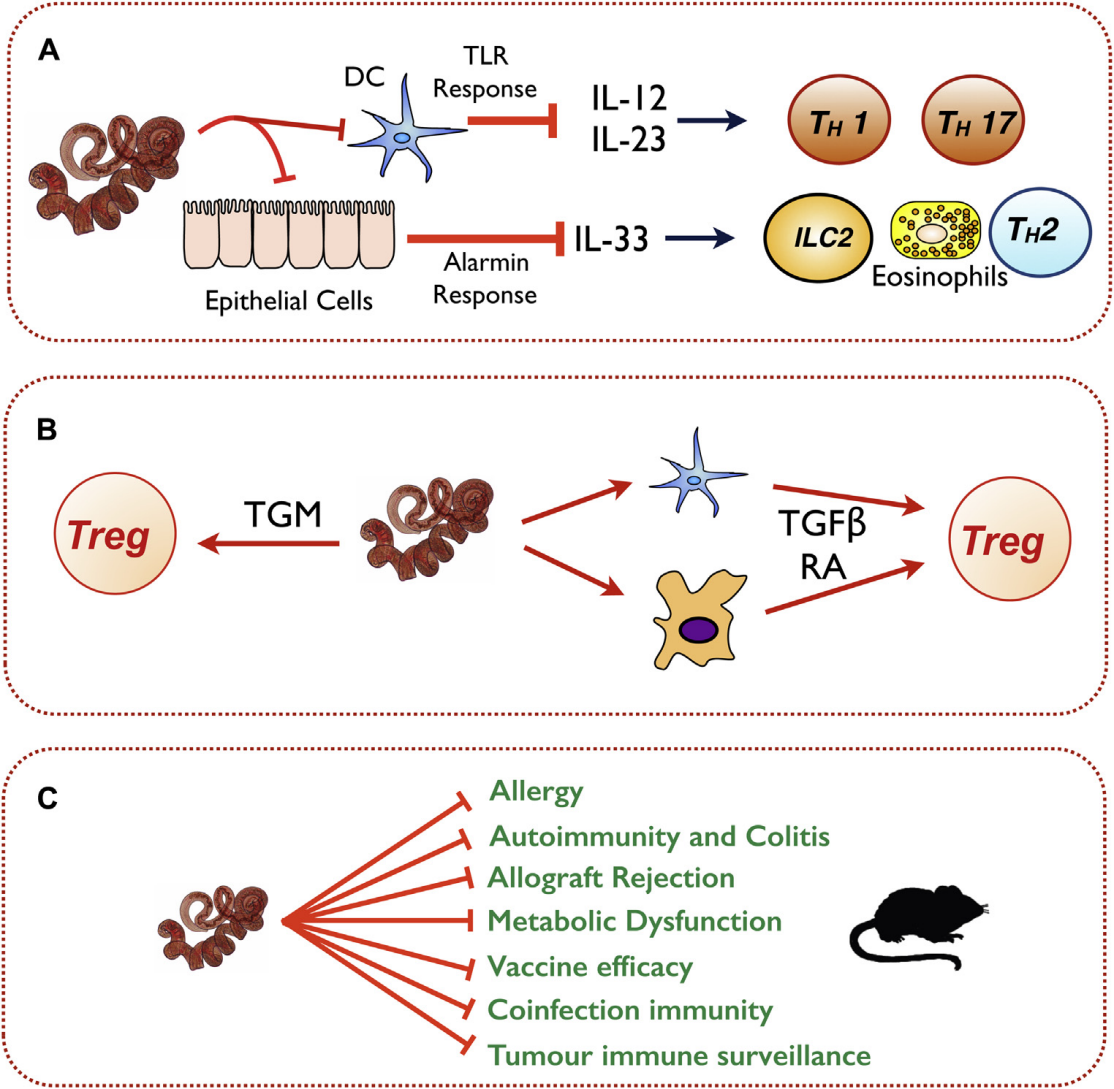
Immune system–parasite interactions during helminth infections. **A,** Blockade of innate sensing and alarmin production, such as inhibiting Toll-like receptor *(TLR)* responses of dendritic cells, thereby impairing inflammatory T_H_1/T_H_17 development, and abrogating epithelial cell production of IL-33, thereby pre-empting the type 2 response. *ILC2*, Type 2 innate lymphoid cell. **B,** Modulation of the adaptive immune response, promoting Treg cell differentiation either directly through production of TGF-β–like mimics *(TGM)* or indirectly by inducing host TGF-β and retinoic acid *(RA)* from DCs and macrophages. **C,** Modification of bystander immune responses in the infected host.

A schematic summary of some known helminth modulatory pathways and targets for immune modulation is presented in Fig 1, while a more detailed list of immunomodulatory effects of parasite products with potential for use in immune-mediated disease is shown in Table I.^45^, ^46^, ^73^, ^103^, ^104^, ^105^, ^106^, ^107^, ^108^, ^109^, ^110^, ^111^, ^112^, ^113^, ^114^, ^115^

As we elucidate mechanisms of immune-mediated parasite ejection, we might appreciate new targets for immunomodulation. For instance, eosinophils are required for ejection of many parasites,^116^ and eosinophil accumulation is potently suppressed by many parasite products in models of allergy.^103^, ^104^, ^117^, ^118^, ^119^ However, as yet, no parasite products have been identified that act directly on this population. In subsequent sections we will propose other likely targets of parasite immunomodulation.

## Interactions between helminths and microorganisms

Intestinal helminths and those that occupy other mucosal sites, such as the lung, cohabit with a spectrum of microbial organisms.^120^, ^121^, ^122^ The entry of helminth parasites, such as *H polygyrus*, *Trichinella spiralis*, or *Trichuris muris*, into the intestinal tracts of mice significantly perturbs the commensal bacterial populations, with important immunologic and metabolic consequences.^123^, ^124^, ^125^, ^126^ In several studies helminth-infected mice show increase in *Lactobacillus* species colonization, which in the case of *H polygyrus* correlates with increased numbers of Treg cells; moreover, prior administration of lactobacilli to mice renders them more susceptible to *H polygyrus* infection, demonstrating a reciprocally beneficial interaction between the metazoan and microbial species.^124^ Moreover, the transfer of intestinal contents from infected to uninfected mice conferred immunomodulatory effects that reduced allergic reactivity in naive recipients.^127^ A mechanistic insight into how the microbiota might favor parasite establishment was gained recently in studies of retinoic acid–related orphan receptor γt–dependent T cells in the gut, which in response to microbial stimulation differentiate to both T_H_17 effectors and retinoic acid–related orphan receptor γt–positive Treg cells, which together repress T_H_2 immunity to *H polygyrus*.^128^ It is presently unknown whether these changes in the commensal population represent an adaptation of commensals to the environment in helminth infection or are due to active modulation by helminth-secreted factors (eg, secreted lysozymes).^120^

## Helminths and homeostasis

Increasingly, regulation of metabolism and weight control is recognized as an immunologic process,^129^, ^130^ and hence it is fascinating that helminth infections can protect against metabolic disorders.^131^ In a seminal study, mice infected with *Nippostrongylus brasiliensis* and fed a high-fat diet were protected against glucose intolerance through activation of adipose tissue eosinophils, which induced alternatively activated M2 macrophages.^132^ Similarly, not only *S mansoni* infection of mice but also administration of soluble antigens from schistosome eggs, expanded adipose eosinophils and M2 macrophages.^133^ Interestingly, one of the major molecular components of soluble antigens from schistosome eggs, ω-1, can itself protect against metabolic disorders when administered to mice,^105^ opening up a biochemical pathway that helminths can activate during infection that proves beneficial to the host.

## Helminths and cancer

There are many parallels between immune responses that result in the progression of tumors and maintenance of parasite infection. By better understanding immunosuppressed responses to parasites and how these could be abrogated to expel the pathogen, we can better understand anticancer responses and how to bolster them for immunity. Parasitic infection could increase carcinogenesis through associated low-grade chronic inflammatory responses (in the absence of parasite ejection), secretion of directly procarcinogenic factors, or suppression of immune surveillance.^134^, ^135^

*Schistosoma haematobium* infection results in deposition of eggs in the bladder wall and is strongly linked to the development of bladder cancer.^136^ Mouse models using egg injection into the bladder wall have shown that egg deposition results in an inflammatory environment, leading to a preneoplastic environment^136^ and predisposing to tumorigenesis. In contrast, the trematode *Opisthorchis viverrini* resides in the bile duct and secretes a granulin-like growth factor (Ov-GRN-1) that directly causes proliferation of host cells and, with cofactors such as dietary carcinogens, leads to transformation of bile duct cells and ultimately cholangiocarcinoma.^137^, ^138^ Likewise, the closely-related parasite *Clonorchis sinensis* also encodes a granulin-like molecule that is hypothesized to carry out the same function.^139^ Because these parasites feed on bile duct cells, it has been proposed that by encouraging cell proliferation, they are decreasing the damage caused by their feeding (while increasing their food source), with carcinogenesis being an unintended byproduct of this pathway.^140^

The least well-studied mechanism of carcinogenesis by parasite infection is suppression of immune surveillance, leading to escape of mutated host cells, which would normally be eliminated by the immune system. Myeloid-derived suppressor cells accumulate during parasitic infections and are either involved in parasite ejection^141^, ^142^ or suppress antiparasite immune responses,^143^ depending on the parasite species and chronicity of infection. In antitumor responses myeloid-derived suppressor cells are a well-characterized suppressive population.^144^ Likewise, the expansion of Treg cells and alternative activation of macrophages during parasitic infection is associated with suppression of antitumor immune responses.^145^ Together, parasitic infection appears to result in a protumorigenic immune milieu. Epidemiologic data in this area are presently lacking, and the effects of parasitic infection in cancer progression requires further attention.

## Epithelial responses

The importance of epithelial barriers in initiation of immune responses is now widely appreciated.^146^ In response to parasitic infection or allergen administration, epithelial cell damage results in release of damage-associated molecular patterns, such as ATP, high mobility group box 1 (HMGB1), uric acid, and S100, as well as proallergic alarmin cytokines, such as IL-33, IL-25, and thymic stromal lymphopoietin, together with more generally inflammatory cytokines, such as GM-CSF and IL-1α.^147^ The critical nature of these responses can be seen in systems in which radioresistant stromal cells (including epithelial cells) are specifically targeted for knockdown of pattern recognition or cytokine receptors in which allergic responses do not develop.^148^, ^149^ Furthermore, because the gut epithelium is critically involved in ejection of parasitic infections (through increased mucus production and epithelial cell turnover), the importance of the epithelium to the antiparasite immune response cannot be overstated.^150^ Combined with the intimate association between many helminths and the epithelial barrier, this makes the epithelium a prime site for helminth modulation.

Release of the alarmin cytokine IL-33 is a potent signal for type 2 response initiation. Mice lacking the IL-33 signaling pathway have abrogated type 2 responses to allergens and parasites and are more susceptible to infection with *Litomosoides sigmodontis*,^151^ *N brasiliensis*,^152^ and *T spiralis*,^153^ whereas administration of exogenous IL-33 leads to ejection of *H polygyrus*,^154^ *N brasiliensis*,^155^ *Strongyloides venezuelensis*,^156^ and *T muris*.^157^ Thus the IL-33 pathway appears to be an ideal target for parasite immunomodulation to allow persistence of infection. Indeed, the excretory/secretory products of *H polygyrus* potently inhibit the IL-33 pathway, both by suppressing IL-33 release^103^ and by suppressing expression of the IL-33 receptor,^158^ resulting in reduced type 2 responses and abrogated inflammation in a mouse model of asthma. Whether these pathways of immunomodulation are common to many intestinal helminths or unique to the chronically infective *H polygyrus* remains to be investigated.

In parallel to IL-33, IL-25 activates type 2 innate lymphoid cells, potentiates type 2 immune responses, and is crucial for ejection of parasites. Mice deficient in IL-25 or its receptor show increased susceptibility to *H polygyrus*^159^, ^160^ and *N brasiliensis* primary or secondary infections.^161^ Chemosensory tuft cells of the intestinal epithelium were recently identified as the major source of IL-25 in the intestine during parasite infection.^162^, ^163^, ^164^ Remarkably, tuft cell–deficient mice show extremely abrogated immunity to *N brasiliensis* infection, with all tuft cell–deficient mice retaining productive infections up to 42 days after infection,^162^ whereas IL-25–deficient mice show only a slightly delayed response.^161^ Thus although tuft cells clearly have an important role in producing IL-25, their antiparasite functions must extend beyond this. As an emerging crucial element of the antiparasite response, it appears likely that some parasites will have developed mechanisms for modulating tuft cell responses to allow their persistence in the host.

## Helminth vaccines

As we have gained greater understanding of the complex response required for ejection of parasites, we have attempted to apply this to development of vaccines against helminth infections. However, to date, no vaccines have been developed for human helminth infections. The reasons for this include the subtle and complex interplay of factors required for ejection,^159^ the potential for collateral damage to the host, the risk of anaphylaxis in infected populations,^165^ and the lack of defined single immunodominant antigens.^166^ Finally, the competing immune regulatory response, which is known to suppress bystander vaccinations,^20^ means that vaccination may only be a successful strategy in populations cured of helminth infections by anthelmintics or in previously helminth-naive children.

## Conclusion

Parasites are subtle but powerful regulators of host immune responses, suppressing some pathways of immune activation (eg, DC antigen presentation, T-cell cytokine and B-cell antibody production, and epithelial cell alarmin release), modulating other pathways (eg, T_H_ cell subset differentiation and B-cell isotype switching), and inducing still others (eg, Treg and Breg cell differentiation and tolerogenic DC responses). The immune pathways required for induction, expansion, and maintenance of antiparasite responses are still being elucidated. As we discover more about how productive antiparasite responses are produced, we are also discovering new pathways for immunomodulation of these pathways by helminth infections and exploring new possibilities for exploiting parasite molecules as therapies for inflammatory diseases.

**What is unknown?**
• What are the molecules secreted by parasites to induce Treg and Breg cells?
• Do multiple parasite products suppress DC responses through ω-1–like ribonuclease activity? Is this unique to schistosomes? Which other mechanisms are co-opted by other parasites?
• Do parasite infections lead to reduced tumor immune surveillance and increased cancer diagnoses?
• Do parasites interfere in epithelial cell responses beyond IL-33?
• How can parasite-derived molecules be used to treat immunopathologies?

## Figures and Tables

**Table I:** Selected parasite-derived molecules with activity in immune-mediated diseases

Immune modulatory effect	Example parasite product	Mechanism of action	Disease models in which efficacy is shown	References
Suppression of innate and adaptive immune cell activation	*A viteae* ES-62	Nonconventional signaling through TLR4, leading to sequestration of PKC-α	Asthma, atopic dermatitis, SLE, and arthritis	106, 107, 108
Suppression of antigen presentation	*S mansoni* ω-1	Degradation of DC mRNA, preventing IL-12 secretion	NOD diabetes, metabolic homeostasis	73, 105, 109
	*F hepatica* FhHDM-1	Inhibition of vacuolar ATPase resulting in reduced endolysosomal acidification	Sepsis	110, 111
	Cystatins: *A viteae* Av17 (AvCystatin), *B malayi* Bm-CPI-2, *N brasiliensis* Nippocystatin	Inhibition of cysteine proteases required for antigen presentation; induction of IL-10 through signaling events downstream of an unknown receptor (Av17)	Asthma, colitis	104, 112, 113, 114, 115
Suppression of ILC2 responses	*H polygyrus* HES	Suppression of ILC2-inducing IL-33 responses	Asthma	103
Induction of Treg cells	*H polygyrus* HES	Secreted TGF-β mimic ligates host TGF-β receptor	Asthma	45
	*S mansoni* SEA/ω-1	Induction of tolerogenic DCs, which produce TGF-β and RA	Type I diabetes	46, 109

*Bm-CPI-2*, *Brugia malayi* cysteine protease inhibitor 2; *FhHDM-1*, *Fasciola hepatica* helminth defense molecule 1; *HES*, *H polygyrus* excretory secretory products; *Hp-CPI*, *H polygyrus* cysteine protease inhibitor; *ILC2*, type 2 innate lymphoid cell; *PKC*, protein kinase C; *RA*, retinoic acid; *SEA*, *Schistosoma mansoni* soluble egg antigen; *SLE*, systemic lupus erythematosus; *TLR*, Toll-like receptor.
